# 

**Published:** 1894-01-27

**Authors:** 


**Notice to Advertisers and Subscribers.**

All Advertisements, Orders for Copies of THE HOSPITAL, and Business Communications should be addressed to The Publisher, *not to the Editor*, THE HOSPITAL, 428, Strand, W.C.

The Cost of Subscription, post free, is as follows:—Per week, 2½d.; per quarter, 2s. 8d.; per half-year, 5s. 3d.; per annum, 10s. 6d Foreign:—Per Annum, 12s. 8d.; payable, in all cases, in advance.

**OUR NEW OFFICES.**

428, Strand, W.C., will henceforth be the Office of this Journal.

**Notices of Births, Deaths, and Marriages** are inserted in this column at a charge of 2s. 6d. for an announcement not exceeding four lines.

### NOTICE TO CORRESPONDENTS.

All MS., Letters, Books for Review, and other matters intended for the Editor should be addressed THE EDITOR, THE LODGE, PORCHESTER SQUARE, LONDON, W.

The Editor cannot undertake to return rejected MS., even when accompanied by stamped directed envelope.

**"Burdett's Hospital Annual," 1893.**

Fourth year of publication. 700 pp. Boards, gilt lettering. A most complete guide to British, Colonial, Indian, and American Hospitals, Dispensaries, Nursing and Convalescent Institutions, and Asylums. Price 5s. Published by THE SCIENTIFIC PRESS (Limited), 428, Strand, W.C.

**NOTICE.—The Index for Vol. XIV. (ending Sept. 30th, 1893) is on sale at the Office of this Journal, price 2½d., post free.**

### SCRAPS AND GLEANINGS.

IT is proposed to further extend the accommodation afforded by the Faversham Infectious Diseases Hospital.

THE annual ball at Chichester, held in favour of the funds of the infirmary, took place recently, at which the attendance numbered 160.

THE late Sir George Elvey, organist to the Queen and to St. George's Chapel, Windsor, has bequeathed £1,000 to the North Cambridgeshire Cottage Hospital.

THE Sanitary Authority of Upton-on-Severn has withdrawn from uniting in the project of the joint injectious diseases hospital with Malvern and Malvern Link.

DURING the present year a new Swiss Official Pharmacopœia is to be issued. It will be in force throughout the whole of Switzerland, excepting the Glarus Canton.

A CONCERT on behalf of the convalescent home in connection with the Hospital Society was held recently at 11, Hill Street, W., by permission of the Duke and Duchess of Newcastle.

"THE Ordnance Survey" Department, County Clare, has forwarded a sum of money collected amongst the men as a small Christmas gift towards the funds of the Clare County Infirmary.

DR. THORNE THORNE, speaking recently at the Sanitary Institute, remarked on the great advance of the death-rate from diphtheria, which he stated to be at present three times as great as in 1872.

A SOCIETY, quite in accordance with the aims of the age, has been started in Berlin for the healthy education of youth. Many of the committee are medical men, and one, Dr. Schwaebe, is the president.

THE sixtieth birthday of the celebrated biologist, Professor Haeckel, of Jena, will be celebrated by the presentation of his bust to the Zoological Institute of that University by his pupils and admirers.

THE official seal of the Bromley Joint Hospital Board has just been affixed to the contract with Mr. T. W. Graty for alterations and additions to the hospital, and also to the bond of £500 with the treasurer of the board.

THE Executive Committee of the Society for the Protection of Animals from Vivisection have also recently transmitted to the Viceroy and members of the Executive Council a protest against the establishment of a Pasteur Institute in India.

SUBSCRIBERS and friends of the Plymouth Royal Eye Infirmary held the 72nd annual meeting of the institution recently. The treasurer's report showed that the legacies and donations were more numerous than the previous year; the subscriptions, however, show a slight decrease.

THE third annual ball in aid of the Bingley Cottage Hospital was held recently at Bingley. This festivity has proved, so far as held, financially, a success to the funds of the institution, and this one in question has been no exception to the rule, but is the most successful so far of the series.

AT the annual meeting of the Northern Counties Hospital for Incurables, held recently, the Board of Management were able to congratulate the subscribers upon the satisfactory results of a year's quiet, steady work, and to present a balance-sheet which would compare favourably with its predecessors.

AT this institution several large items of expenditure have been met, the furnishing of the large extensions at Walmersley, and a lift for the patients, as well as a new apparatus for the supply of hot water at Mauldeth.

THE second annual report of the Children's Hospital at Leicester was of a very gratifying character. It stated that the amount raised was not so large as the previous year, but was yet very satisfactory. The number of children collected during the year was [illegible]80. The total receipts towards the expenses had been £500.

THE gift from "Two Friends" of £1,000 towards the funds of the committee of the "Consumptive Homes for Scotland" will enable the authorities to commence the building of an engine and the washing-house and offices connected with the group of homes contemplated for erection for the treatment of that disease.

THE Eastern Dispensary, Bath, had its annual meeting recently. During the year the working of the institute had been, it was stated, carried on in a satisfactory manner. The number of patients registered for treatment, 4,696, had been equal to the average of recent years. A balance of £179 remained on the treasurer's accounts.

AT the thirty-fourth annual meeting of the governors and subscribers to the Birmingham Dental Hospital the General Committee stated that the structural alterations at the hospital had been completed at a cost of nearly £500. The Surgical Committee reported that 18,467 operations had been performed during the year. The financial statement for the year showed an adverse balance of £106.

### BOOKS RECEIVED.

REEVES AND TURNER.
"A Legal Handbook for the Use of Hospital Authorities." By L. S. Bristowe.

**Magazines and Pamphlets.**—The Humanitarian. Toilers of the Deep. The Sewage Disposal of Isolated Dwellings. The Zenana. The Sun. Service for the King. Reich's Medicinal Anzeiger. The Gentlewoman. The English Illustrated Magazine. London. In His Name. Invention. Provincial Medical Journal. Monthly Homœopathic Review. The Asclepiad. The Indian Medico-Chirurgical Review. Public Health. Guy's Hospital Gazette. Nursing Notes. The Health Record. The Child's Guardian.

### Medical Vacancies and Appointments.

### APPOINTMENTS.

R. H. Brew, L.R.C.P., L.R.C.S.Edin., appointed Medical Officer and Public Vaccinator for the Chew Magna District of the Clutton Union. T. Frankish, M.B., C.M., and B.Sc.Edin., appointed Resident Medical Officer to the National Sanatorium for Consumption and Diseases of the Chest, Bournemouth. A. H. Godwin, M.R.C.S., L.R.C.P.Lond., appointed House-Surgeon to the Wallasey Dispensary. J. Griffith, M.R.C.S. and L.R.C.P., appointed Clinical Assistant to the Royal Westminster Ophthalmic Hospital. A. R. Kocke Hudson, late District Medical Officer for the Upper Widnes District of the Prescot Union, appointed Medical Officer to the Anglo-Chilian Railway and Nitrate Company (Limited), Jocopiela, Chile; Medical Officer to the Compania Saletrera de "Santa Fé," Joco; Medical Officer to Messrs. Sloman, Donah, and Co., Oficina "Bueno Esperanza," Joco; and Medical Officer to the Nitrate Railways, Joco. F. H. Marson, M.B., B.S.Durh., M.R.C.S., L.R.C.P., appointed House Surgeon to the Staffordshire General Infirmary. J. G. Modlin, M.B., B.S.Dunelm, L.S.A.Lond., appointed Medical Officer for the Monkwearmouth East District of the Sunderland Union. J. R. Preston, M.R.C.S.Eng., L.R.C.P. Lond., appointed House-Surgeon to the Jessop Hospital for Women, Sheffield. P. J. Probyn, M.R.C.S. and L.R.C.P., appointed Clinical Assistant to the Royal Westminster Ophthalmic Hospital. F. C. Wallis, M.B.Cantab, F.R.C.S., appointed Surgeon to the Orthopædic Department of Charing Cross Hospital. L. J. Weatherbe, M.B. and C.M.Edin., appointed Medical Officer for the Kimberworth District of the Rotherham Union. A. O. Wiley, L.R.C.P.Edin., L.R.C.S.I., appointed Medical Officer of the Workhouse of the Knaresborough Union. E. Arthur Gibson, M.B., C.M., Glasgow, House Surgeon to the Paddington Green Children's Hospital.

### VACANCIES.

### Resident Medical Officers.

Central London Ophthalmic Hospital, Gray's Inn Road, W.C. House Surgeon. Applications, with testimonials, to the Secretary, by February 5th.

Buckinghamshire General Infirmary, Aylesbury. Resident Surgeon and Apothecary, unmarried and duly qualified. Salary £80, increasing £10 per annum to £100, with board, lodging, and washing. Applications and testimonials to Mr. George Fell, Solicitor, Aylesbury, by January 29th.

### Non-Resident Medical Officers.

Hospital for Sick Children, Great Ormond Street, Bloomsbury, W.C. Assistant House Surgeon. Appointment for six months. Salary £20. Applications, with not more than three testimonials, to the Secretary, by Tuesday, January 30th.

Royal Pimlico Dispensary, Buckingham Palace Road, S.W. Attending Medical Officer; must reside in the district. Applications and testimonials to the Secretary, by February 5th.

Swindon New Town Local Board. Medical Officer of Health. Must hold D.P.H. certificate. Salary £100 per annum. Applications to Henry Kennier, Clerk to the Board, by January 29th.

### EDINBURGH TRIPLE QUALIFICATION.

### Papers Recently Set.

FINAL EXAMINATION.

Questions in Medical Jurisprudence and Hygiene. (Only Three Questions to be answered, including Nos. 3 and 4.)

1. The body of a newly-born infant has been found with marks of violence upon it, and a certain woman is suspected of the infanticide. What examination of the woman should be made to prove or disprove the suspicion, and how would you proceed to her examination?

2. What legal points are involved in connection with (1) the "dying declaration" of a man who has been criminally assaulted; (2) the use of notes by a medical witness in a Court of Law; (3) the form of Medical Certificates, and more especially the form of a Certificate of Lunacy?

3. What symptoms in a Patient would lead you to suspect Chronic Irritant Poisoning? State how you would detect the presence of Arsenic or Antimony in the vomit, and what steps you would take to prevent the continued administration of the poison.

4. What Diseases are classed as "Zymotic"? In what Acts of Parliament are they dealt with, and how? What are the usual means of disinfection of clothing?

Demy 8vo., 200 pp., cloth extra, price 3s. 6d.,

**SURGICAL WARD-WORK AND NURSING**: A practical Manual of Clinical Instruction for Nurses and Students. This book will be found especially useful to beginners, to whom its simple description of elementary principles and treatment in surgery will render it invaluable. By ALEXANDER MILES, M.D. (Edin.), C.M., F.R.C.S.E. Over one hundred illustrations.

LIST OF CHAPTERS.

SECTION I.—ANTISEPTIC SURGERY. Chap. 1. General Principles of Antiseptic Surgery—2. Antiseptic Lotions—3. Antiseptic Powders and Unguents—4. Materials for Dressings—5. Lotion Basins—6. Ward Table and Dressing Tray—7. Antiseptic Dressing—8. A Spray Dressing—9. Management of Surgical operations—10. The Lotion Table—11. Dressings Table—Anæsthetics—12. Preparation of Patient for Operation—13. Treatment after Operation—14. Nursing of Special Cases after Operation.

SECTION II.—THE USE OF REST IN SURGERY. 15. Extension Apparatus—16. Plaster and Starch Cases—17. Appliances for Joint Affections—18. Appliances for Fractured Bones.

SECTION III.—BANDAGING. 19. Bandaging—20. Special Bandages—21. Special Bandages (*continued*)—22. Special Bandages (*continued*).

SECTION IV.—SURGICAL INSTRUMENTS AND APPLIANCES. 23. General Instruments—24. Instruments for Hæmorrhage—25. Knives—26. Saws—27. Bone Instruments—28. Instruments used in Trephining—29. Mouth Instruments, etc.—30. Intestinal Instruments—31. Urethral Instruments—32. Lithotomy and Lithotrity Instruments—33. Laryngeal Instruments—34. Aural, Nasal, and Ophthalmic Instruments—35. Gynæcological Instruments.

"Will furnish much-needed guidance to the student and the nurse in the early stages of their apprenticeship."—*Times.*

"This is a fully illustrated handbook for the use of junior students of medicine and nurses, which should prove of very great value, Dr. Miles being unquestionably an authority on the subject of which he writes."—*Publishers' Circular.*

Just ready, Important New Text-Book of Ophthalmic Nursing.

Crown 8vo., cloth gilt, pp. 200, profusely illustrated, and with many woodcuts, price 3s. 6d., post free,

**OPHTHALMIC NURSING.** By SYDNEY STEPHENSON, M.B., F.R.C.S.E., Surgeon to the Ophthalmic School, Hanwell, W., &c., &c. A complete text-book on this branch of nursing, illustrated by numbers of original figures, and containing a glossary of terms and list and description of instruments used in ophthalmic nursing.

As yet no book has been published dealing with nursing in diseases of the eye. As a text-book, "Ophthalmic Nursing" will be invaluable to nurses desiring to qualify in this particular branch of nursing. The managers of Ophthalmic Institutions are invited to adopt this work as a text-book for their nursing staffs. The book contains a frontispiece, "Vertical Section of the Eye-ball and Eye-lids," and sixty-one other illustrations It is concisely yet exhaustively written, and its method of treatment and style will render it especially serviceable to the Nursing Profession.

THE LANCET says: "The work now before us gives the result of his experience to those who have charge of Ophthalmic patients, and will prove useful. A good account is given of the proper arrangements and preparations that should be made for operations, with the various modes of bandaging one or both eyes. The work may very advantageously be studied by nurses and clinical clerks, or dressers who are about to enter the Ophthalmic service of a hospital."

NURSING NOTES says: "A book on Ophthalmic Nursing has been a real need which is now well supplied. . . . . Those contemplating going in for Ophthalmic Nursing should study this useful work. . . . . One of the most useful books for nurses we have seen for a long time."

THE OPHTHALMIC REVIEW says: "The author has evidently endeavoured to write in language which will be intelligible to nurses of all grades, and we congratulate him on the clearness with which his statements are expressed. . . . . Chapters II. and III., upon 'The Germ Theory of Disease' and 'Contagion and Infection,' are good, and the latter contains useful rules for the avoidance by nurses and others of the contagion of eye disease."

"Will prove of inestimable value."—*Publishers' Circular.*

**London: THE SCIENTIFIC PRESS (Limited), 428, Strand, W.C.**

London: THE SCIENTIFIC PRESS (Limited), 428, Strand, W.C.

### ACCOUNT BOOKS FOR INSTITUTIONS.

Designed by HENRY C. BURDETT.

Ruled in accordance with the uniform system of accounts advocated by the Metropolitan Hospital Sunday Fund, and prepared for the convenience and assistance of Secretaries who present annual returns for participation in the Hospital Sunday Fund Grants.

These Books are ruled so that the various descriptions of Receipts and Expenditure, &c., may be entered uniformly under the special headings and in the columns prepared for them.

There are TWO SERIES, No. 1 being for the larger Institutions, and No. 2 for Cottage Hospitals and Small Institutions. The sets are sold complete, at a specially reduced price; or each book may be had separately, at the prices set out below:—

### SERIES No. 1.—FOR HOSPITALS AND INSTITUTIONS.

(Any of these Books may be purchased separately; a reduction is made if the complete set is taken.)

1. ANALYSIS JOURNAL. Royal, 350 pp. Divided into seven sections, with parchment tags. Headings as follows:—
   A. MAINTENANCE.
   1. Provisions. 5. Rent.
   2. Surgery & Dispensary. 6. Salaries.
   3. Domestic. 7. Miscellaneous Expenses
   4. Establishment Charges.
   B. ADMINISTRATATION.
   1. Management. 2. Finance.
   (Uniform with Headings in Income and Expenditure Table.) Each Heading is ruled with pages containing 15 columns. Price £3.
2. CASH BOOK. Foolscap, 300 pp., printed Headings, specially ruled. Price £1.
3. CASH ANALYSIS AND RECEIPT BOOK. Foolscap, extra width, 300 pp., ruled, with 15 columns, printed Headings identical with Income and Expenditure Table. Price £1 5s.
4. SECRETARY'S PETTY CASH BOOK. 300 pp., foolscap, ruled with 13 columns, printed Headings identical with Income and Expenditure Table. Price £1.
5. LIST OR REGISTER OF ANNUAL SUBSCRIBERS. Double foolscap, 300 pp., ruled with 10 columns, printed Headings for the years 1893-1900 inclusive. Price £1 15s.
6. ALPHABETICAL REGISTER BOOK. Foolscap, 300 pp., ruled, with date, name and address, and cash colums, cut into alphabetical sections, with totals at end for annual balances. Price £1 6s.
7. SUBSCRIPTION REGISTER. Foolscap, ruled with 8 columns, printed Headings for date when subscriptions become due, name and address, and cash columns for years 1893 to 1898 inclusive. Price £1 1s.
8. LINEN REGISTER. Foolscap long quarto, 300 pp., ruled with columns for Stock, Condemned, Remaining, and Issued Linen. also total in Ward and Remarks, also printed lists of Hospital Linen. Price 8s. 6d.
9. INCOME AND EXPENDITURE TABLE. Basis of uniform system. For yearly balances and statements. Royal. Price 6d. per sheet, or 5s. per dozen.
10. SPECIAL APPEAL ACCOUNT TABLE. Foolscap. Price 3d. per sheet, or 2s. 6d. per dozen.

PRICE OF SET COMPLETE, £10 NET.

### SERIES No. 2.—FOR COTTAGE HOSPITALS AND SMALLER INSTITUTIONS.

1. CASH ANALYSIS, RECEIPT, AND EXPENDITURE BOOK. Double foolscap, 300 pp., ruled with 18 columns, printed Headings identical with Income and Expenditure Table, specially prepared for Cottage Hospitals and Small Institutions. Price £1 15s.
2. SECRETARY'S PETTY CASH BOOK.—300 pp., foolscap, ruled with 13 columns, printed headings identical with Income and Expenditure Table. Price £1.
3. INCOME AND EXPENDITURE TABLE. Basis of uniform system. For yearly balances and statements. Royal. Price 6d. per sheet, or 5s. per dozen.
4. LINEN REGISTER. Foolscap, 300 pp., ruled with columns for Stock, Condemned, Remaining, and Issued Linen, also total in Ward and Remarks, also printed lists of Hospital Linen. Price 8s. 6d.
5. LIST OR REGISTER OF ANNUAL SUBSCRIBERS. Foolscap. 300 pp., ruled with columns for the years 1893-1900 inclusive. Price £1.
6. SPECIAL APPEAL ACCOUNT TABLES. Foolscap. Price 3d. per sheet, or 2s. 6d. per dozen.

PRICE OF SET COMPLETE, £4 NET.

The books are all uniformly bound in half basil, with gilt letterings, and are ruled on best paper, and in every way prepared for practical use at the offices of Institutions.

### ACCOUNTS, AUDIT, AND TENDERS; the Uniform System of,

For Hospitals and Institutions, with Certain Checks upon Expenditure. Also the Index of Classification. Compiled by a Committee of Hospital Secretaries, and adopted by a General Meeting of the same, 18th January, 1892. And certain Tender and other Forms for securing Economy. By HENRY C. BURDETT. Demy 8vo, cloth, 6s.

This is a hand-book to the account books, explaining their nature and uses, the method of keeping them, and the general principles upon which they were designed.

All having to do with hospital accounts will find much valuable information in this work.

Secretaries making up returns for participation in the Donations of the Metropolitan Hospital Sunday Fund will find it invaluable. It will be of special interest, also, to the officials of all Institutions—Treasurers, Secretaries, and Auditors—as it expounds practically the Uniform System, which is now being widely adopted as a means of saving labour and of bringing all systems of accounts into uniformity.

"This useful and practical little book. No one interested in the hospital question can treat with indifference anything which falls from Mr. Burdett. . . . A uniform system of keeping their accounts. . . . Would be a boon to hospitals generally."—*British Medical Journal.*

London: THE SCIENTIFIC PRESS (Limited), 428, Strand, W.C.

THE FEEDING OF INVALIDS.

Now Ready. Demy 8vo., cloth gilt, pp. 260. Price 3s. 6d.

**ART OF FEEDING THE INVALID.** By a Medical Practitioner and a Lady Professor of Cookery.

A book that the probationer can place in the hands of the nurse as a guide in his absence.

Matrons of institutions, and all engaged in the care of the sick, have found it invaluble as a daily guide.

This important work fully and clearly describes the nature and tendency of diseases most frequently met with, and gives a general outline upon which the diet of the invalid should be regulated. Corresponding with the chapters are lists of new and original recipes, which are intended for daily use and reference.

The labour and anxiety of providing suitable diet for invalids will, by the use of this book, be reduced to a minimum.

Among the subjects treated are the following :—The Feeding of Invalids generally ; Food ; Food in Acute Febrile Diseases, in Convalescence and Chronic Illness, in Indigestion, in Gout, in Constipation and Diarrhœa, in Liver and Kidney Diseases, in Diabetes, in Tuberculosis and Consumption, in Corpulence, and for Children.

"The Art of Feeding the Invalid" has the approval of Members of the Faculty, its Medical author being widely known as the author of a standard text-book.

The Recipes are entirely new, having been especially written by a Professional Cook who holds diplomas and has had vast experience as a lecturer.

---

To be ready shortly. Royal 8vo., 500 pp., 60 illustrations, cloth gilt. Price One Guinea.

**HOSPITALS, DISPENSARIES, AND NURSING:** Being Transactions of Section III. International Congress of Charities, Correction, and Philanthropy, held in Chicago, 1893.

This valuable collection of papers will be published in one handsome volume as above, and comprises a variety of subjects, each of which is dealt with by a representative and leading authority.

Out of the total of nearly a hundred authors may be mentioned the names of Miss Florence Nightingale, Dr. J. S. Billings, U.S.A., Miss Isabel Hampton, Mr. Henry C. Burdett, Mrs. Dacre Craven, Miss Louisa Twining, Miss Lückes, and many others.

The edition will be limited, and the pages are not stereotyped.

As sole English publishers of the work, "The Scientific Press" are now booking subscriptions for early copies of the work, which will be delivered on the day of publication.

---

**IMPORTANT NEW HAND-BOOK OF MIDWIFERY.**

18*mo., profusely illustrated with original cuts, tables, &c.; about* 260 *pp., handsomely bound in leather, Price* 6s.

**A PRACTICAL HAND-BOOK OF MIDWIFERY.** By Francis W. N. Haultain, M.D.

This Hand-book is produced in a portable and convenient form for reference, and is intended mainly as a practical help to the busy practitioner. It is bound in roan, of a size convenient for the pocket.

To Students it brings forward succinctly the chief points of practical importance in the study of Obstetrics.

THE CHAPTERS ARE AS FOLLOWS:

1. Diagnosis of Pregnancy.
2. Pathology of Pregnancy.
3. Diseases due to Pregnancy.
4. Abnormal Pregnancy.
5. Extra Uterine Gestation.
6. Premature Expulsion, etc.
7. Hygiene of Pregnancy and calculation of date of Confinement.
8. Labour.
9. The Puerperium.
10. The Puerperium (*cont.*)
11. Laborious Labour.
12. Delay in Second Stage.
13. Contracted Pelvis.
14. Preternatural Labour.
15. Pelvic Presentations.
16. Transverse Presentations.
17. Version, or Turning.
18. Instrumental Labour.
19. Embryalcia.
20. Induction of Premature Labour.
21. Cæsarian Section.
22. Complicated Labour.
23. Post Partum Hæmorrhage.
24. Rupture of Uterum.
25. Complex Labour.
26. Minor Complication of the Puerperium.
27. Grave Complications of Puerperium.
28. Inflammatory Affections of Puerperium.
29. Puerperal Insanity.
30. Artificial Feeding of Infants.

LIST OF ILLUSTRATIONS.

FIG.
1. Diagram showing points of greatest intensity of Fœtal Heart sounds.
2. Diagram of Fœtal Head.
3. Do. do. from above.
4. Normal Pelvis (Brim).
5. Do. (Outlet).
6. Flat Pelvis.
7. Malacosteon Pelvis.
8. Naegele's Pelvis.
9. Roberts' Pelvis.
10. Method of Delivery of Extended Head in Breech Cases.
11. Delivery by "Prague Seizure."
12. Simpson's Axis Traction Forceps (Murray's Modification).
13. Perforator.
14. Braun's Cranioclast.
15. Hick's Cephalotribe.
16. Simpson's Basilyst.
17. Diagram of Transverse Horizontal Section through Pelvis, showing Normal Seats of Pelvic Cellular Tissue.

**Price Six Shillings (post free).**

---

London: THE SCIENTIFIC PRESS (Limited), 428, STRAND, W.C.

In the press, fifth year of publication, about 750 pp., crown 8vo., cloth gilt, price 5s.,

### BURDETT'S HOSPITAL AND CHARITIES ANNUAL,

**Being the Year Book of Philanthropy for 1894.**

Containing a Review of the Position and Requirements of the Voluntary Charities, and an Exhaustive Record of Hospital Work for the Year. It will also be found to be the most Useful and Reliable Guide to British, Colonial, and American Hospitals, Dispensaries, Nursing and Convalescent Institutions and Asylums.

Edited by HENRY C. BURDETT.

The new volume for 1894 will bear evidence of many improvements. The work has been entirely re-set in new type specially founded, and the whole of the lists of institutions and the particulars relating to them have been so carefully revised as to justify the belief that the volume will be of the greatest use to all interested in or connected with the institutions.

Many additions have been made, the sections dealing with the General Charities and with American and Colonial Institutions having been considerably extended. And the special articles are of more than ordinary interest and importance.

Orders are now being booked for subscription copies, to be delivered on the day of publication, and should be addressed without delay to the publishers at the undermentioned address.

CONTENTS OF SPECIAL CHAPTERS.

| | |
|---|---|
| Chapter I. | The Year 1893. |
| Chapter II. | A Central Board of Medical Charities. |
| Chapter III. | The Cost of Hospital Management. |
| Chapter IV. | The Cost of Hospital In-Patients and Maintenance. |
| Chapter V. | Our Philanthropic Institutions; Character and Cost of the Work Done. |
| Chapter VI. | The Income of Voluntary Hospitals in 1892. |
| Chapter VII. | Hospital Expenditure in 1892. |
| Chapter VIII. | Hospital Cost, Income, and Expenditure in the United States. |
| Chapter IX. | The Uniform System of Accounts for Hospitals and Charities of all Grades; Book-keeping, Accounts, Audit, Tenders. |
| Chapter X. | Practical Hints on the Construction of Hospitals for the Sick and Insane, Orphanages, and Charitable Buildings, generally, and a few words on Drainage, Internal Fittings, Appliances, and Furniture. New Buildings in 1893. |
| Chapter XI. | What In-Patients Provide for Themselves. |
| Chapter XII. | Hospital Sunday Organisations. |
| Chapter XIII. | Hospital Saturday Organisations, |
| Chapter XIV. | The Proportion of In and Out Patients to Population, and the Chief Questions Involved. |
| Chapter XV. | Nurses and Nursing at Home and Abroad. |
| Chapter XVI. | The Duty of the Governors of Charities in Relation to an Adequate Provision for their Officials and Employés. |
| Chapter XVII. | Dispensing. |

London: THE SCIENTIFIC PRESS (Limited), 428, Strand.

---

JUST PUBLISHED. 8vo, 3s. 6d., post free.

### LEGAL HANDBOOK for the use of HOSPITAL AUTHORITIES.

By LEONARD SYER BRISTOWE, M.A., Oxon, Barrister-at-Law.

REEVES and TURNER, 100, Chancery Lane, W.C.

